# Dynamic Connectivity Analysis Using Adaptive Window Size

**DOI:** 10.3390/s22145162

**Published:** 2022-07-10

**Authors:** Zoran Šverko, Miroslav Vrankic, Saša Vlahinić, Peter Rogelj

**Affiliations:** 1 Department of Automation and Electronics, Faculty of Engineering, University of Rijeka, 51000 Rijeka, Croatia; zsverko@riteh.hr (Z.Š.); sasa.vlahinic@riteh.hr (S.V.); 2 Faculty of Mathematics, Natural Sciences and Information Technologies, University of Primorska, 6000 Koper, Slovenia; peter.rogelj@upr.si

**Keywords:** brain connectivity analysis, brain network dynamics, complex Pearson correlation coefficients

## Abstract

In this paper, we propose a new method to study and evaluate the time-varying brain network dynamics. The proposed *RICI-imCPCC* method (relative intersection of confidence intervals for the imaginary component of the complex Pearson correlation coefficient) is based on an adaptive window size and the imaginary part of the complex Pearson correlation coefficient. It reduces the weaknesses of the existing method of constant sliding window analysis with narrow and wide windows. These are the low temporal precision and low reliability for short connectivity periods for wide windows, and high susceptibility to noise for narrow windows, all resulting in low estimation accuracy. The proposed method overcomes these shortcomings by dynamically adjusting the window width using the *RICI* rule, which is based on the statistical properties of the area around the observed sample. In this paper, we compare the *RICI-imCPCC* with the existing constant sliding window analysis method and describe its advantages. First, the mathematical principles are established. Then, the comparison between the existing and the proposed method using synthetic and real electroencephalography (*EEG*) data is presented. The results show that the proposed *RICI-imCPCC* method has improved temporal resolution and estimation accuracy compared to the existing method and is less affected by the noise. The estimation error energy calculated for the *RICI-imCPCC* method on synthetic signals was lower by a factor of 1.22 compared to the error of the constant sliding window analysis using narrow window size *imCPCC*, by a factor of 2.87 compared to using wide window size *imCPCC*, by a factor of 6.69 compared to using narrow window size *wPLI*, and by a factor of 4.72 compared to using wide window size *wPLI*. Analysis of the real signals shows the ability of the proposed method to detect a *P300* response and to detect a decrease in dynamic connectivity due to desynchronization and blockage of mu-rhythms.

## 1. Introduction

Recently, various monitoring methods have been used to detect the dynamics of brain networks [1,2]. Theoretical considerations and empirical observations of humans, macaques, and rats using various recording methods, such as *fMRI* [3,4,5,6,7], blood-oxygenation-level-dependent functional magnetic resonance imaging (*BOLD-fMRI*) [8,9], *MEG* [10], and *EEG* [11,12,13], have been established and suggest that connectivity is time-dependent, dynamic, and is associated with rhythmic activity [11,14,15,16].

In this study, electroencephalography (*EEG*) was used to monitor neuron activity. *EEG* is a monitoring method that records the electrical activity of the brain [17]; i.e., it measures the voltage fluctuations emanating from connected neurons. This method allows non-invasive detection of neuron interactions/connections with high temporal resolution [18].

In general, brain connectivity analysis is divided into: structural and functional. Structural connectivity analysis studies a huge map of anatomical connections [19]. The most appropriate recording methods for structural connectivity analysis are magnetic resonance imaging (*MRI*) [20], functional magnetic resonance imaging (*fMRI*) [21], and diffusion tensor imaging (*DTI*) [22,23].

In addition, functional connectivity analysis provides information about the interactions between distant brain regions [11]. Generally, there are two types: undirected (estimating the degree of connectivity) and directed (estimating the degree and direction of connectivity), and in this paper we focus on undirected connectivity. The second division of functional connectivity deals with static and dynamic connectivity analysis, and in this paper we focus on dynamic connectivity analysis. The most appropriate recording methods for analyzing functional connectivity are magnetoencephalography (*MEG*) and electroencephalography (*EEG*), due to their high temporal resolution.

Static functional connectivity indices always assumes that the degree of connectivity remains constant throughout the observation period [11]. Accordingly, these methods are only able to estimate an average degree of connectivity over the observation period. A variety of static connectivity indices are used in research, but the most commonly used indices are the phase locking value (*PLV*) [24,25] and the weighted phase lag index (*wPLI*) [26]. The primary difference between these two indices is capacity to bypass the influence of volume conduction.

In [27], an alternative to *PLV* and *wPLI* is proposed, called the complex Pearson correlation coefficient (*CPCC*). It is a single measure that provides information about the connectivity components with and without the influence of volume conduction. The imaginary part of the complex Pearson correlation coefficient (*imCPCC*) is closely related to the *wPLI* measure, and the absolute value of the complex Pearson correlation coefficient (*absCPCC*) is closely related to the *PLV*. If we compare *imCPCC* with *wPLI* and *PLV* with *absCPCC*, we see that the difference is mainly in the normalization of the measures to the interval [0,1].

All of the static indices of functional connectivity described above cannot detect the periods when brain regions are functionally connected or not. The need to obtain additional information about when the changes in functional connectivity occur brings us to the study of dynamic functional connectivity methods.

The most commonly used method to study the dynamics of brain networks is sliding window analysis [11,12,13,28]. In this method, the index of functional connectivity is calculated in a window with predefined number of samples (*N*), and then the window is moved to the next set of samples with or without overlap. Functional connectivity (*FC*) is calculated for an interval of interest.

In [29], constant sliding window analysis is used to analyze the differences between 30 patients with early Parkinson’s with mild cognitive impairment and 37 patients with early Parkinson’s without mild cognitive impairment. In [29], window lengths were set to 500 to 2000 data points (samples) with a step of 10 samples. The overlap between windows was also set to 0–250 data points. In the end, 906 different window lengths were determined for four subbands, and the window length that showed the largest significant difference between the observed patient groups was selected as the optimal sliding window. In [30], dynamic functional connectivity was calculated using the sliding constant window analysis method with window lengths of 45, 60, 75, and 90 s to confirm their hypothesis. The 75 s window size was chosen because the most relationships were found with it. The higher-order dynamic functional connectivity time series were computed in [31] using the constant sliding window analysis method. In [32], the *wPLI* was computed based on predefined 2 s epochs, and constant sliding window analysis was proposed to extract information about connectivity patterns in the future, focusing on determining the tradeoff between temporal resolution and estimation error, i.e., determining the optimal window size. In [33], it is mentioned that the window size should be short enough to represent a good tradeoff between the ability to capture dynamic connectivity and sensitivity to noise. The dynamic functional connectivity is currently not widely used because of the limitations of currently used methods.

The shortcomings of constant sliding window analysis are poor temporal resolution at wide window size and results being affected by noise at narrow window size.

In other words, the overall variability of the estimate *FC* using sliding windows increases as the size of the interval decreases [28] and vice versa. Thus, to obtain more reliable estimates, we would like to have the *FC* indices computed on as wide an interval as possible without including parts of the signal with different statistical properties.

**Figure 1:**
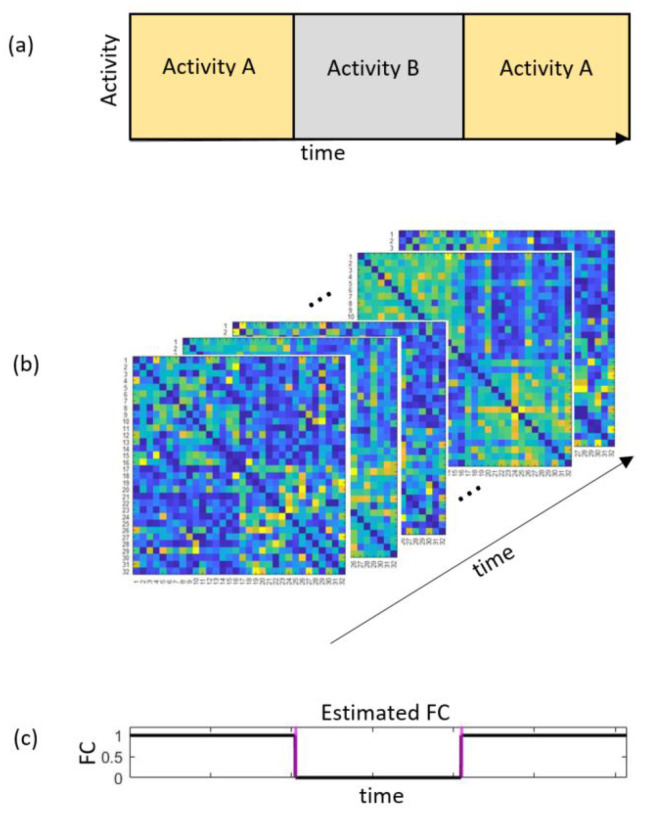
Illustration of the problem to be solved by the proposed method. (**a**) The experimental paradigm with the sequence of different activities. (**b**) The connectivity matrix estimated for each time sample. (**c**) The expected estimation of the functional connectivity index (*FC*) for a selected electrode pair. The estimated *FC* must reflect the activities in time.

In order to obtain such intervals that allow us to detect the time periods when brain regions are functionally connected or not with good temporal resolution and without the influence of noise on the estimation results, we propose to use an adaptive modification of a statistical method called the intersection of confidence intervals (*ICI*) rule [34,35,36] called the relative *ICI* (*RICI*) algorithm [37]. The *RICI* method will allow us to obtain results with better temporal resolution and will allow less influence of noise on the estimates of the values of the *FC* indices. Figure 1 illustrates the idea of dynamic functional connectivity analysis.

The rest of the article is organized as follows. In Section 2, we propose a novel method for estimating the dynamics of the brain using *RICI* algorithm and the *imCPCC* measure. In Section 3, we illustrate brain dynamics in practical experiments with synthetic and real *EEG* signals. We compare the sliding window analysis method with the new proposed method. The paper ends with a discussion and a conclusion.

## 2. Methods

We have recognized the shortcomings of existing methods in their choices of window width, such as the inability to detect periods with functional connectivity and periods without functional connectivity at wide window sizes, i.e., low temporal resolution, and when using a narrow window, the influence of noise on the estimated *FC* values. In this paper we propose a method to alleviate these problems. We name it *RICI-CPCC*, which stands for the relative intersection of confidence intervals for the imaginary component of the complex Pearson correlation coefficient estimation method, and it adopts previously proposed solutions [27,37]. The first part of our solution is the use of the *RICI* algorithm, which gives us the advantage of a variable window width and is used to estimate functional connectivity. The second part of our solution is the use of the *CPCC*, whose advantage is the simultaneous evaluation of the connectivity with and without the influence of the volume conduction and the use of a uniform scaling. We can calculate the *CPCC* for each individual sample and obtain high temporal resolution but low connectivity resolution due to high noise. Oppositely, we get poor temporal resolution and high connectivity resolution using wide windows. The optimal window width depends on the signals themselves, as the low temporal resolution is due to joining signal parts with different statistical properties, and the low connectivity resolution is due to the low attenuation to noise at small window sizes. To find the optimal window width that gives us good temporal resolution and is not affected by noise, we introduce a variable window width obtained by the *RICI* algorithm. The pseudocode of the *RICI* algorithm used to estimate the time-varying functional connectivity index is shown in Algorithm 1.
**Algorithm 1:** The *RICI* algorithm used to estimate the time-varying *FC*.1:**for***each time sample***do**2:    *Set the minimal window size*3:    **while** $R\geq R_{c}$ **do**4:        *Compute confidence interval (CI) for a given window*5:        *Estimate lower and upper confidence interval boundaries* $D_{u}$ *and* $D_{l}$6:        *Update* $D_{u_{MIN}}$ *and* $D_{l_{MAX}}$7:        *Compute new criterion ratio R*8:    **end**9:    *Compute functional connectivity for the given window*10:**end**

Any estimation procedure of a window size for dynamic analysis can be performed in two ways: parametrically and non-parametrically. Parametric estimators expect a priori information about the probability density functions (*PDFs*) of signal and noise. If these assumptions are correct, parametric estimators provide more accurate estimates than nonparametric estimators. In most practical applications, as with *EEG* analysis, a priori information is not available, so nonparametric estimators must be used.

Accordingly, in this section we have defined the estimation methods based on the goodness-of-fit statistic, the intersection of confidence intervals (*ICI*) rule, and the improved method, the relative *ICI* (*RICI*) algorithm.

### 2.1. Measuring Temporal Functional Connectivity

Temporal functional connectivity (*TFC*) means that the functional connectivity index is calculated in a predefined interval. We monitor brain network dynamics using the *CPCC* (complex Pearson correlation coefficient) *FC* index, introduced in [27]. The *CPCC* measure can be split into two components (measures): the absolute value of the complex Pearson correlation coefficient (*absCPCC*) and the imaginary component of the complex Pearson correlation coefficient (*imCPCC*). In [27], the relationships between the most commonly used methods of functional connectivity, phase locking value (*PLV*) and weighted phase lag index with *absCPCC*, and *imCPCC* are analytically and numerically proven. *PLV* can be replaced by *absCPCC* and *wPLI* by *imCPCC*, it was concluded [27]. *CPCC* is defined as a Pearson’s linear correlation coefficient:

(1) $$CPCC\left(x_{a1},x_{a2}\right)=\frac{\sum_{n=1}^{N}x_{a1,n}\cdot x_{a2,n}^{*}}{\sqrt{\sum_{n=1}^{N}{\left|x_{a1,n}\right|}^{2}}\cdot \sqrt{\sum_{n=1}^{N}{\left|x_{a2,n}\right|}^{2}}},$$

where *N* is the number of samples, $x_{a1}$ and $x_{a2}$ are analytical signals given by the Hilbert transform, and ${\left\{.\right\}}^{*}$ is the complex conjugate operator.

In the following, we will use *imCPCC*, which is defined as follows:

(2) $$imCPCC_{x_{a1},x_{a2}}=\left|Im\left[r\left(x_{a1},x_{a2}\right)\right]\right|.$$

**Figure 2:**
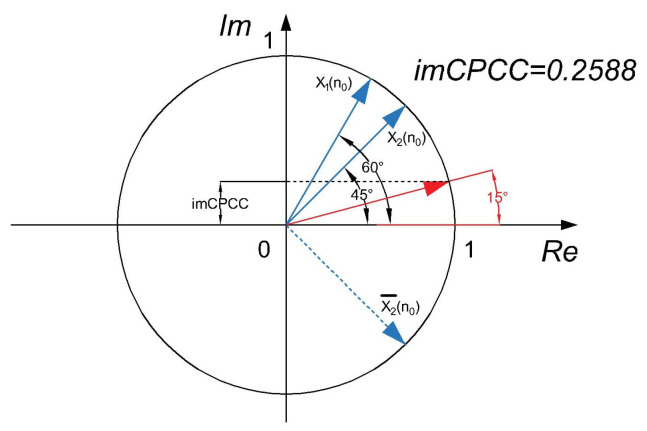
Visualization of the calculation of *imCPCC* for a sample.

The calculation of *imCPCC* for a sample can be illustrated by creating two unit vectors, each belonging to a sample of the observed signals. The scalar product of the first vector and the conjugate complex value of the second vector are divided by the product of their magnitudes; see Figure 2.

### 2.2. Using the RICI Method to Determine the Optimum Window Width

The *ICI* rule [38,39] is based on a number of estimates of a observed parameter or in our example, *FC* index. The estimation procedure is based on different window sizes. If we define a set of windows with growing size as:

(3) $$H=\{h_{k}|h_{k}>h_{k-1},k=1,2,...,K\},$$

where $h_{k-1}$ is a subset of $h_{k}$. The *K* estimates of *FC* are computed for *K* consecutive windows. Furthermore, we can define confidence intervals for the estimated functional connectivity index $\hat{FC}$ (i.e., in this example, for the estimated value of *imCPCC*) as follows:

(4) $$D_{k}=\left[{\hat{FC}}_{h_{k}}\left(n_{0}\right)-\Gamma \sigma_{h_{k}}\left(n_{0}\right),{\hat{FC}}_{h_{k}}\left(n_{0}\right)+\Gamma \sigma_{h_{k}}\left(n_{0}\right)\right],$$

where $n_{0}$ is the signal sample under consideration and $\sigma_{h_{k}}\left(n_{0}\right)$ stands for the standard deviation of the estimated signal sample on the observed window. The width of the confidence interval is defined with the empirically set constant Γ. This leads to the introduction of confidence intervals; the upper $D_{u}$ and lower $D_{l}$ boundaries of the confidence interval are defined as follows:

(5) $$D_{u}\left(n_{0}+\Delta n\right)=\hat{FC}\left(n_{0}+\Delta n\right)+\Gamma \sigma \left(n_{0}+\Delta n\right),$$

(6) $$D_{l}\left(n_{0}+\Delta n\right)=\hat{FC}\left(n_{0}+\Delta n\right)-\Gamma \sigma \left(n_{0}+\Delta n\right).$$

Δn represents the observation window length in a range $\left[0,N-n_{0}\right]$ for the forward calculation (where *N* is the total number of samples) and in a range $\left[0,n_{0}\right]$ for the backward calculation of the confidence intervals intersection. The *ICI* algorithm yields the number of samples suitable for calculating *FC*. The largest Δn satisfies the following condition:

(7) $$D_{u_{MIN}}\left(n_{0}+\Delta n\right)\geq D_{l_{MAX}}\left(n_{0}+\Delta n\right),$$

where $D_{u_{MIN}}$ and $D_{l_{MAX}}$ are defined as follows:

(8) $$D_{u_{MIN}}\left(n_{0}+\Delta n\right)=min\left(D_{u}\left(n_{0}\right),...,D_{u}\left(n_{0}+\Delta n\right)\right),$$

(9) $$D_{l_{MAX}}\left(n_{0}+\Delta n\right)=max\left(D_{l}\left(n_{0}\right),...,D_{l}\left(n_{0}+\Delta n\right)\right).$$

The optimal window size for the considered signal sample is defined as:

(10) $$h\left(n_{0}\right)=\Delta n_{F}+\Delta n_{B}+1,$$

where $\Delta n_{F}$ is the number of samples obtained from the forward calculation and $\Delta n_{B}$ is the number of samples obtained from the backward calculation.

**Figure 3:**
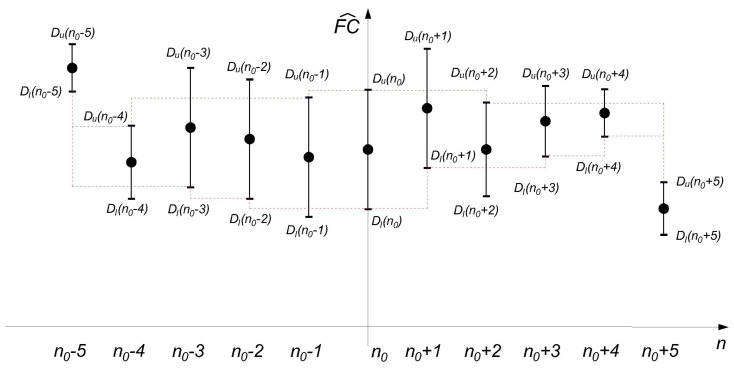
An example of an intersection of confidence intervals. The blue dashed line marks $D_{u_{MIN}}$, and the red dashed line marks $D_{l_{MAX}}$. If the lines cross, the condition (Equation (7)) is not met. For the largest width of the window for the observed sample, the last one that satisfies the condition is then taken.

An example of intersection of confidence intervals (*ICI*), with the lengths of the confidence intervals is shown in the Figure 3.

The *ICI* algorithm depends on the value of the parameter Γ [37,38]. Large values of Γ lead to over-smoothing of the estimated values, and small values of Γ lead to under-smoothing. This deficiency can be addressed using cross-validation or the Γ variable selection method [38], but these methods require additional repetitions of the *ICI* algorithm and therefore take additional time.

To avoid this deficiency, an improved version of the *ICI* algorithm was developed, called the relative intersection of confidence intervals (*RICI*) [37].

The *RICI* algorithm [37] solves the problem of dependence on Γ parameter. Based on the overlap of two back-to-back confidence intervals, a new parameter called the relative amount of the overlapping confidence intervals was defined as follows:

(11) $$R\left(n_{0}+\Delta n\right)=\frac{D_{u_{MIN}}\left(n_{0}+\Delta n\right)-D_{l_{MAX}}\left(n_{0}+\Delta n\right)}{D_{u}\left(n_{0}+\Delta n\right)-D_{l}\left(n_{0}+\Delta n\right)},$$

where $D_{u_{MIN}}$ and $D_{l_{MAX}}$ are the minimum value of the upper boundaries and the maximum value of the lower boundaries defined by Equations (8) and (9). $D_{u}$ and $D_{l}$ are the upper and lower boundaries of the last observed confidence intervals. The new optimal window size $h(n_{0})$ for the observed sample is determined as the largest Δn that satisfies the condition called the *RICI* rule, defined as follows:

(12) $$R\left(n_{0}+\Delta n\right)\geq R_{C},$$

where $R_{C}\in \left[0,1\right]$ is the predetermined threshold introduced by the *RICI* algorithm [37]. If we choose $R_{C}$ equal to 0, the *RICI* algorithm will revert to the *ICI* algorithm.

**Figure 4:**
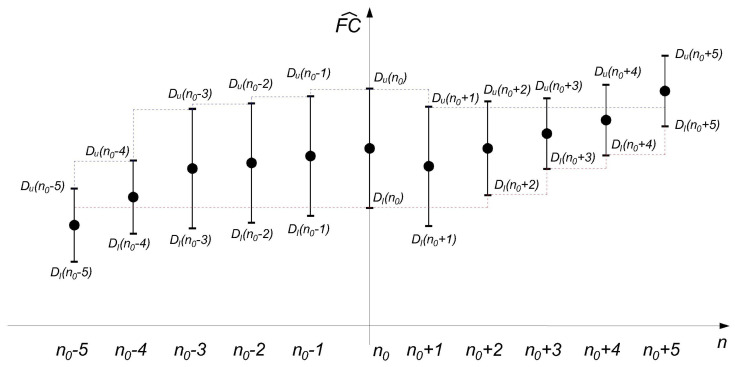
An example of the relative intersection of confidence intervals *RICI*, with $R_{C}=0.5$. The blue dashed line marks $D_{u_{MIN}}$, and the red dashed line marks $D_{l_{MAX}}$. If the $R(n_{0}+\Delta n)$ falls below the value $R_{C}$ according to the condition (Equation (12)), the condition is not satisfied. For the largest width of the window for the observed sample, the last one that satisfies the condition is then taken.

An example of the relative intersection of confidence intervals, with the lengths of the confidence intervals, is shown in the Figure 4.

### 2.3. Description of Datasets

In this subsection we describe the datasets used in our article.

#### 2.3.1. Description of Synthetic Signals

We generated two synthetic sinusoidal signals with a center frequency of 10 Hz and a sampling frequency of 256 Hz; 3072 samples or 12 s of signals were generated. In the range [1,1024], signal two leads signal one by +π/2, and conversely, in the range [2049,3072], signal two is lagging signal one by −π/2. From 1025 to 2048, the two observed signals are in phase. These two signals could be considered as a pair of electrode signals.

In a second stage of synthetic signal analysis, noise with a normal distribution and a standard deviation of 0.1 is added to signal one, and noise with a normal distribution and a standard deviation of 0.3 is added to signal two.

#### 2.3.2. Description of the Auditory Oddball Dataset

The auditory oddball dataset [40] used consists of four EEG recordings from two healthy subjects. Two recordings were obtained while subjects were not under hypnosis and two while subjects were under hypnosis. In this article, we have used only the two recordings in which the subjects were not under hypnosis. Subject S01 was a male right-handed person and subject S02 was a female right-handed person. *EEG* recordings were obtained using 27 EEG active g.tec electrodes (F8, F4, Fz, F3, F7, FC6, FC2, FC1, FC5, T8, C4, Cz, C3, T7, TP10, CP6, CP2, CP1, CP5, TP9, P8, P4, Pz, P3, P7, O2, O1) and a recording device. The participant sat in a comfortable reclining chair. The participant listened to the tones through in-ear headphones (E-A-RTONE Gold, Auditory Systems, Indianapolis, IN, USA). The participant was instructed to listen to a stream of tones and count the occurrences of the odd (low) tones. Frequent (high) tones occurred between the odd tones. The participant was asked to count only the odd tones. *EEG* recordings were obtained with a sampling rate of 512 Hz, a bandpass filter of 0.01–100 Hz, and a notch filter at 50 Hz. Stimulus-onset asynchrony (*SOA*) was 900 ms.

#### 2.3.3. Description of the Motor Imagery Dataset

Ten subjects participated in the experiment [41]: six men and four women, ranging in age from 21.4 to 28 years, all of whom were right-handed. Each of them sat in a comfortable chair, and the *LCD* monitor was placed about 1 m in front of their eyes. *EEG* recordings were made with an electrode cap (Easycap) and with *Ag/AgCl* electrodes placed on the scalp according to the international 10/20 system. Three electrodes were used: C3, Cz, and C4. *EEG* recordings were obtained with a sampling rate of 250 Hz, a bandpass filter of 0.5–100 Hz, and a notch filter at 50 Hz. There are the results of several feedback experiments in this dataset, but for the purposes of this article, we used results on imagined movement of the right hand. The experiment began with the crosshairs fixed in the center of the screen and a brief audible warning tone (1 kHz, 70 ms) lasting a total of 3 s. A visual cue was then presented for 1.25 s. Subsequently, subjects imagined right hand movements for the next 4 s. Each trial ended with a short pause of at least 1.5 s. The dataset consists of sixty trials of imagining right hand movement.

### 2.4. Offline Preprocessing

**Figure 5:**
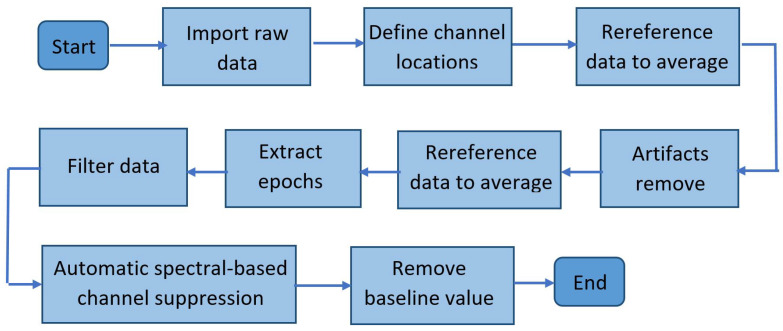
The illustration of the offline preprocessing steps performed prior to dynamic connectivity analysis.

Offline preprocessing of the real raw data described in Section 2.3.2 and Section 2.3.3 was performed according to the steps in the flowchart shown in Figure 5.

Raw *EEG* data were imported into *Matlab* using the *EEGLab* toolbox, and channel positions were defined in the software. In addition, the data were rereferenced to the average. Then, the data were filtered to the desired frequency bands, and automatic spectral-based channel suppression (z=5) was performed using the *EEGLab* *“pop-rejchan”* function. In addition, artifacts were removed using the *EEGLab IClabel* plugin, thresholds to remove components greater than or equal to 0.9 for artifacts and less than or equal to 0.05 for brain activity were selected, and data were rereferenced to the average. The epoch was extracted, and the baseline value was removed.

## 3. Results

In this section, we compare the proposed *RICI-imCPCC* estimation with the most commonly used method: sliding window analysis with constant window width using *wPLI* [26] and *imCPCC*.

### 3.1. Synthetic Signals

**Figure 6:**
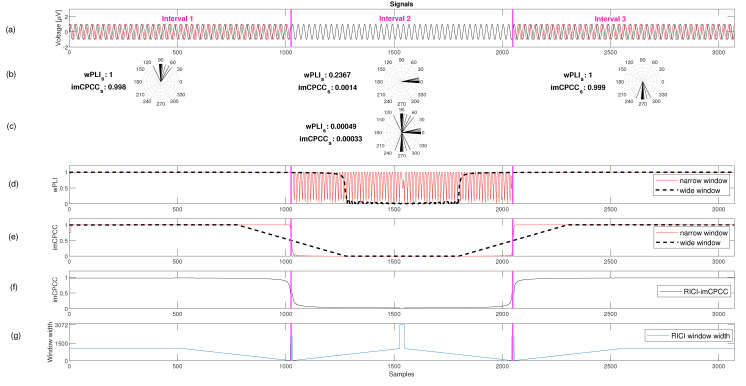
An example of a *RICI-imCPCC* estimation procedure for the ideal synthetic signals ($R_{C}=0.8$ [37]. For an example, the 95% confidence interval was obtained for Γ=1.96 [38,39]). (**a**) Two synthetic sinusoidal signals considered as a pair of electrode signals. The phase angle difference between these signals is different in three intervals separated by magenta vertical lines. (**b**) The calculated temporal functional connectivity of *wPLI* and *imCPCC* for each of the intervals separately with predefined interval boundaries. In addition, this line shows the distribution of the unit vector phase angle differences in the polar domain. (**c**) This gives us an insight into the static functional connectivity *imCPCC* value calculated for the entire signal period and the distribution of unit vector phase angle differences in the polar domain. (**d**,**e**) The estimated *wPLI* and *imCPCC* values calculated using the sliding constant window analysis method with narrow (window size equal to 10 samples) and wide (window size equal to 500 samples) windows. (**f**) The estimate using the *RICI-imCPCC* method, and (**g**) the change in window width for each observed sample defined using the *RICI-imCPCC* method.

We calculated the dynamics of the brain using the proposed *RICI-imCPCC* and the most commonly used constant sliding window analysis method. The graphs representing the estimates of functional connectivity are shown in Figure 6. Figure 6a shows the two signals with three intervals of three different behaviors marked with magenta vertical lines. In the first and third intervals, temporal functional connectivity of the *imCPCC* should produce a very high level of connectivity, and the *imCPCC* of the second region should have a very low value.

Observing the static connectivity for the provided signals gives imCPCC≅0, but if we observe the entire signal, the dynamics of the connectivity are not pronounced. The distribution of the unit vector phase angle differences in the polar domain on the entire signal period is shown in the Figure 6c. If we observe each interval separately, we get different values for the *imCPCC* value. The *imCPCC* values for each interval and the distribution of the unit vector phase angle differences in the polar domain are shown in the Figure 6b. For the first interval, the *imCPCC* value is ≅1, for the second interval it is ≅0, and for the third interval it is ≅1. The second interval is an example of volume conduction between two observed signals, and because of the ability of *imCPCC* to avoid the influence of volume conduction, the *imCPCC* value is ≅0.

Figure 6d shows the estimation of the *wPLI* index using the constant sliding window analysis method with narrow and wide window lengths. The use of a narrow window leads to high variability in the second interval. The high variability in the situation where we have a distribution around zero is another reason to use *imCPCC* instead of *wPLI*. In general, using a narrow window leads to an estimate with good temporal resolution, and the opposite for a wide window size.

Estimating the functional connectivity index with a narrow constant window size yields a good result in this ideal situation, since the phase differences have only two main changes during the observation period, and these two changes are fast. Estimating the value of functional connectivity with a constant window size for wide window length leads to incorrect values for *imCPCC* in the samples where changes occur because the rate of change is too fast. In other words, the temporal resolution of the changes is low and the exact time of changes is difficult to estimate; see Figure 6e. With the *RICI-imCPCC* estimation procedure, the *RICI* algorithm defines the window width based on the statistical properties of the phase differences between the observed signals. The estimated values are very similar in this example with a narrow constant window size; see Figure 6f. The change in window width for each observed sample defined by the *RICI-imCPCC* method is shown in Figure 6g.

**Figure 7:**
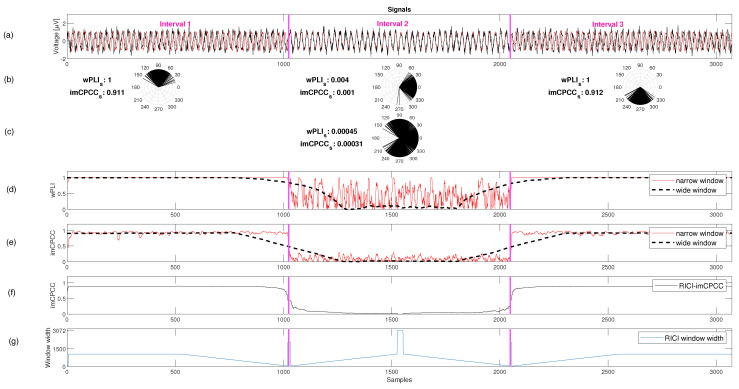
An example of an *RICI-imCPCC* estimation procedure for the noisy synthetic signals ($R_{C}=0.8$ [37]. For an example, the 95% confidence interval was obtained for Γ=1.96 [38,39]). (**a**) Two noisy synthetic sinusoidal signals considered as a pair of electrode signals. The phase angle difference between these signals is different in three intervals separated by magenta vertical lines. (**b**) The calculated temporal functional connectivity of *wPLI* and *imCPCC* for each of the intervals separately with predefined interval boundaries. In addition, this line shows the distribution of the unit vector phase angle differences in the polar domain. (**c**) This gives us an insight into the static functional connectivity *imCPCC* value calculated for the entire signal period and the distribution of unit vector phase angle differences in the polar domain. (**d**,**e**) The estimated *wPLI* and *imCPCC* values calculated using the sliding constant window analysis method with narrow (window size equal to 10 samples) and wide (window size equal to 500 samples) windows. (**f**) The estimate using the *RICI-imCPCC* method, and (**g**) the change in window width for each observed sample defined using the *RICI-imCPCC* method.

Figure 7a shows two sinusoidal synthetic signals as in Figure 6a, but with added noise (superposition). The static functional connectivity *imCPCC* value and the temporal functional connectivity *imCPCC* values on each of the predefined intervals are similar to the results shown in Figure 6; see also Figure 7b,c. Inspection of Figure 7d suggests that the estimated *wPLI* values are affected by noise when a narrow window is used in the second interval, where the distribution of the phase angle differences is around zero. When using a wide window size, temporal resolution and *FC* results are affected by the noise, as can be seen when comparing Figure 6d and Figure 7d. Comparing the estimates for *imCPCC* and *wPLI*, we concluded that *imCPCC* has better temporal resolution for a wide window and lower variability for a narrow window in the second interval. However, the estimated values of the *imCPCC* in the constant sliding window analysis and the proposed *RICI-imCPCC* are quite different. The estimated values generated with a constant narrow window have higher variability (Figure 7e) than the values generated by the proposed *RICI-imCPCC* estimation procedure. Moreover, the smoothness produced by *RICI-imCPCC* is due to the adaptive window width defined by the *RICI* algorithm. *RICI-imCPCC* is not affected by noise; see Figure 7f. In addition, the *RICI-imCPCC* provides good temporal resolution. The variation of the window width over time is also shown in Figure 7g.

For the *FC* estimates shown in Figure 7e,f, the estimation error energy was calculated. If the estimation error is defined as follows:

(13) $$e\left(n\right)=FC\left(n\right)-\hat{FC}\left(n\right),$$

where *FC*(*n*) are the assumed ideal values and $\hat{FC}\left(n\right)$ are the estimated values. The estimation error energy is defined as:

(14) $$E_{e}=\sum_{n=1}^{N}{\left|e\left(n\right)\right|}^{2},$$

where *e*(*n*) is estimation error for each observed time sample. The estimation error energy values for all observed methods are given in Table 1.

From Table 1, we can conclude that the estimation error energy is lowest for the *imCPCC* results estimated by the *RICI-imCPCC* method ($E_{e}=35.72$).

### 3.2. Auditory Oddball Real-Life Signals

In this experiment, we focused on the alpha band, because it is predominant, and alpha connectivity patterns can be different according to different cortical generators depending on the state of the brain [32]. Therefore, the signals were filtered to 8–13 Hz according to [32,42,43,44].

**Figure 8:**
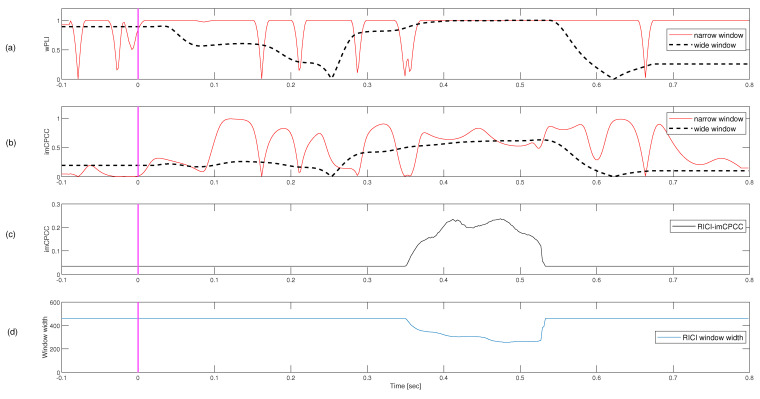
An example of an *RICI-imCPCC* estimation procedure for the real signals ($R_{C}=0.8$ [37], Γ=1.96 [38,39]). The minimum starting window size Np, used in RICI-imCPCC procedure according to the [28] should be the number of samples corresponding to the period of the lowest observed signal frequency, which means: Np=1/8∗512=64 in our example. The growth step of the window is N=1. In this example is observed electrode pair *C4-TP10*. (**a**,**b**) The estimated *wPLI* and *imCPCC* values calculated using the sliding constant window analysis method with narrow (red line, window size is equal to 10 samples) and wide (black dashed line, window size is equal to 128 samples) windows. (**c**) The estimate using the *RICI-imCPCC* method, and (**d**) the change in window width for each observed sample defined using the *RICI-imCPCC* method.

Figure 8a shows estimation of *wPLI* using constant sliding window analysis with wide and narrow window widths (for subject S01). If we expect some changes in the connectivity between three and five hundred milliseconds due to the P300 [42,43], observing *wPLI* estimation with wide window size produces bad temporal resolution. By observing estimation with a narrow window, we can see that the *wPLI* value is almost always equal to one, except for the intervals where the phase angle difference changes sign, and consequently, wPLI approaches zero. Based on the theory of [42,43], during auditory oddball, the electrode signals are not constantly connected, and therefore *wPLI* gives incorrect information. Figure 8b shows estimation of *imCPCC* using constant sliding window analysis with wide and narrow window sizes. Similarly to *imCPCC*, when using narrow window size, the results are affected by the noise, and using wide window size, the results have bad temporal resolution. Both these shortcomings are resolved using the proposed *RICI-imCPCC* method (see Figure 8c), which produce good temporal resolution and is not affected by the noise. Adaptive window sizes are represented in Figure 8d.

**Figure 9:**
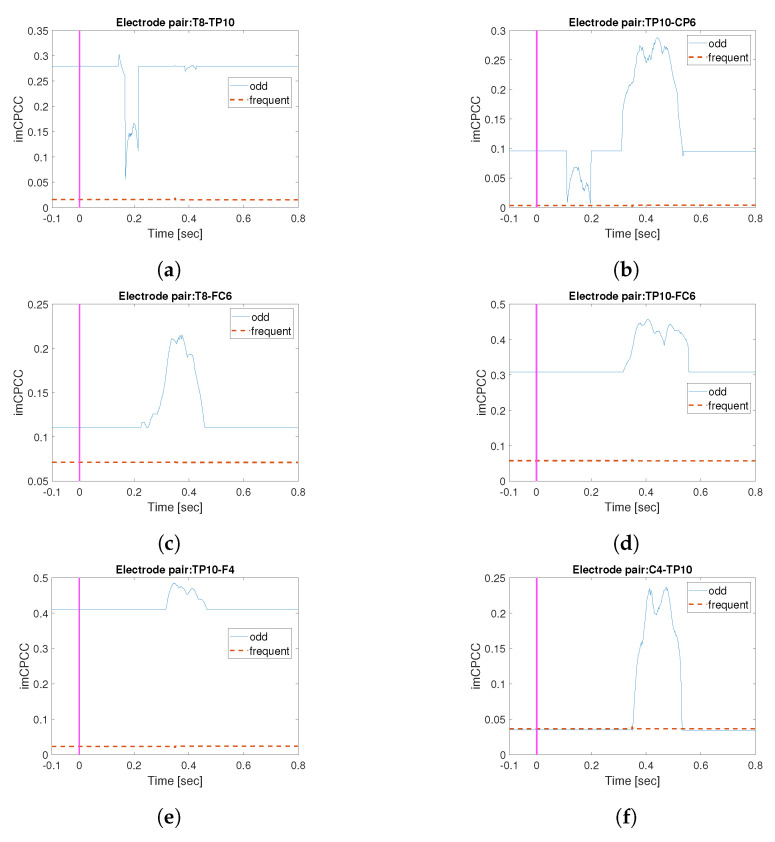
An example of *RICI-imCPCC* estimated values for frequent and odd tones in the observed task ($R_{C}=0.8$ [37], Γ=1.96 [38,39]). The minimum starting window size Np, used in the RICI-imCPCC procedure according to the [28], should be the number of samples corresponding to the period of the lowest observed signal frequency, which means: Np=1/8∗512=64 in our example. The growth step of the window is N=1. The blue line shows the *RICI-imCPCC* estimated values for odd tone, and the red dashed line shows the *RICI-imCPCC* estimated values for frequent tone. Different electrode pairs are shown. (**a**) shows the *RICI-imCPCC* values for the electrode pair T8-TP10, (**b**) for TP10-CP6, (**c**) for T8-FC6, (**d**) for TP10-FC6, (**e**) for TP10-F4, and (**f**) for C4-TP10.

Figure 9 shows the mean *RICI-imCPCC* values (through all 27 trials) for different electrode pairs, for subject *S01*. Marked with the red line are *RICI-imCPCC* results for observation of odd tones, and the blue dashed line shows the same for frequent tones. From Figure 9, we can conclude that the connectivity between electrodes was the same most of the time for frequent tones, whereas the connectivity changed over time for odd tones. The changes in connectivity between T8-TP10, Figure 9a, are due to the participant hearing and processing a tone (areas BA21,22) [45,46]. In addition, information is transmitted to the part of the brain responsible for processing and computation [46], area BA40 [45] (electrode pair TP10-CP6; see Figure 9b). In addition, information from the area responsible for processing complex sounds [46] is transmitted to area BA44 [45], which is responsible for working memory (i.e., short-term memory [46]), electrode pair T8-FC6; see Figure 9c. Another indicator of activation of working memory area BA44 [45] is the change in connectivity between TP10-FC6; see Figure 9d. Area BA08 [45] is responsible for auditory perception and working memory, as indicated by the dynamic connectivity TP10-F4; see Figure 9e. The change in connectivity between TP10-C4 could be due to the wiggling of the fingers [46] during the experiment; see Figure 9f. With this explanation, we would like to emphasize the possibility of practical application of the proposed *RICI-imCPCC* method.

### 3.3. Motor Imagery Real-Life Signals

**Figure 10:**
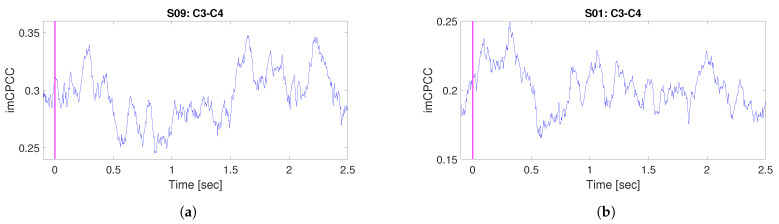
An example of the mean (through 60 trials, frequency band: 8–12 Hz) *RICI-imCPCC* estimated values for imagining right hand movement ($R_{C}=0.8$ [37], Γ=1.96 [38,39]). The minimum starting window size Np used in *RICI-imCPCC* procedure according to the [28] should be the number of samples corresponding to the period of the lowest observed signal frequency, which means: Np=1/13∗250=20 in our example. The growth step of the window was N=1. As a baseline we used −0.1 to 2.5 s. (**a**,**b**) show the *RICI-imCPCC* values for subjects S01 and S09 for the electrode pair C3-C4.

The frequencies of interest are in 8–12 Hz range, which corresponds to the mu-rhythm (the other name is sensorimotor rhythm because it is localized above the sensorimotor strip of the cortex) [47]. Subjects S03 and S10 are discarded in the preprocessing analysis due to a large number of artifacts. Figure 10 shows the dynamic connectivity between the electrode pair C3–C4 for subjects S01 and S09. The sensorimotor rhythm is blocked by the corresponding hand movement or hand touch; accordingly, desynchronization between the electrode pair C3–C4 should be detected during the motor imagery experiment [47]. Connectivity could be interpreted as a measure of the degree of synchronization between some electrode pairs. Accordingly, we expect a decrease in dynamic connectivity when desynchronization between electrode pairs occurs. In Figure 10a,b, the desynchronization between C3 and C4 during the observation of dynamic connectivity is visible in the interval from 0.5 to 0.9 s for subject S01 and in the interval from 0.5 to 1.5 for subject S09. The peak that can be observed immediately after the trigger event and before 0.5 s could be explained as a consequence of the event-related potential (*ERP*).

## 4. Discussion and Conclusions

In this work, we have proposed new methods for monitoring the dynamics of brain networks. We introduced the relative intersection of confidence intervals calculated for the imaginary component of the complex Pearson correlation coefficient (*RICI-imCPCC*). We compared it with the most commonly used method for observing the dynamics of brain networks, constant sliding window analysis.

The most commonly used brain network connectivity indices (*PLV* [24,25], *PLI* [25], *wPLI* [26]) calculate the connectivity value for the entire observed period (static functional connectivity indices). If our observed signal has multiple intervals with different synchronisation properties for the observed electrode pairs and if we consider each interval separately, we get an accurate connectivity index result (if we compute *imCPCC* exactly for the interval of interest, i.e., temporal functional connectivity). The problem, however, is how to identify the start and end times of intervals with different synchronization properties when we do not know in advance the main properties of the observed signal, which is the case for *EEG* signals.

In this work, we have shown that this weakness can be compensated for by using the *RICI* algorithm to define the width of the window and accurately determine the timing of the change in the relationship between the observed pair of electrodes. With *RICI-imCPCC* and the ability to independently define the window width according to the statistical features of the phase angle difference between the two observed signals, we can precisely define the required window width for *imCPCC* estimation using the observed sample, which makes a precise estimate of synchronization or changes in information flow between the observed electrodes or brain regions smoother.

Using the constant sliding window analysis method with a narrow window size results in high variability of estimated index values when synchronization changes are slow. When using the sliding constant window analysis method with a wide window size, it is impossible to estimate at what time the connectivity changes occurred when the changes are fast, i.e., bad temporal resolution. The *RICI-imCPCC* method overcomes both shortcomings because this method can determine the window width according to the *RICI* rule based on the statistical properties of the area around the observed sample. Moreover, unlike the constant sliding window analysis method with a narrow window size, *RICI-imCPCC* is not affected by the noise. *RICI-imCPCC* provides estimation results with high accuracy and good temporal resolution.

In future work, we will use the fast intersection of confidence intervals (*FICI*) method [48], which allows us to estimate connectivity in real time with minimally reduced estimation accuracy. The use of *FICI* could be used to monitor brain network dynamics during neurofeedback protocol and can be used in brain–computer interfaces to interpret difficulties in brain connections during various procedures. The proposed algorithm could also be used as a preprocessing tool for extracting graph-theoretic measures [18] to explore hypotheses about brain dynamics based on these measures.

After the above discussion, we can conclude that the newly proposed *RICI-imCPCC* method can overcome the shortcomings of sliding constant window analysis with both narrow and wide window sizes, and therefore provides us with the opportunity to further investigate the brain network’s dynamics. 

## Figures and Tables

**Table 1:** This table presents the values of the estimation error energy ($E_{e}$) calculated for the values of *FC* shown in Figure 7e,f. The $E_{e}$ values were calculated for *wPLI* and *imCPCC* estimated values obtained using the constant sliding window analysis method with narrow and wide window sizes and the *RICI-imCPCC* estimation method.

Methods	$E_{e}$
Narrow window size-*wPLI*	239.13
Wide window size-*wPLI*	168.81
Narrow window size-*imCPCC*	43.91
Wide window size-*imCPCC*	102.73
*RICI-imCPCC*	35.72

## Data Availability

Not applicable.

## Abbreviations

The following abbreviations are used in this manuscript:

absCPCC	absolute value of complex Pearson correlation coefficient
BOLD-fMRI	blood-oxygenation-level-dependent functional magnetic resonance imaging
CPCC	Complex Pearson correlation coefficient
DTI	Difussion Tensor Imaging
EC	eyes closed
EEG	Electroencephalography
EO	eyes open
ERP	event-related potential
FC	Functional Connectivity
FICI	Fast Intersection of Confidence Intervals
fMRI	Functional Magnetic Resonance Imaging
ICI	Intersection of Confidence Intervals
imCPCC	imaginary component of complex Pearson correlation coefficient
MEG	Magnetoencephalography
MRI	Magnetic Resonance Imaging
PDFs	Probability Density Functions
PLI	Phase Lag Index
PLV	Phase Locking Value
RICI	relative Intersection of Confidence Intervals
RICI-imCPCC	relative Intersection of Confidence Intervals for imaginary component
	of complex Pearson correlation coefficient
TFC	Temporal functional connectivity
wPLI	Weighted Phase Lag Index
